# Ratings of Perceived Exertion and Self-reported Mood State in Response to High Intensity Interval Training. A Crossover Study on the Effect of Chronotype

**DOI:** 10.3389/fpsyg.2017.01232

**Published:** 2017-07-18

**Authors:** Jacopo A. Vitale, Antonio La Torre, Roberto Baldassarre, Maria F. Piacentini, Matteo Bonato

**Affiliations:** ^1^Laboratory of Biological Structures Mechanics, Istituto Ortopedico Galeazzi (IRCCS) Milan, Italy; ^2^Department of Biomedical Sciences for Health, Università degli Studi di Milano Milan, Italy; ^3^Functional Evaluation and Analysis of Sport Performance, Department of Movement, Human and Health Sciences, Foro Italico University of Rome Rome, Italy

**Keywords:** chronotype, POMS, HIIE, mood, physical activity

## Abstract

The aim of this study was to investigate the influence of chronotype on mood state and ratings of perceived exertion (RPE) before and in response to acute high intensity interval exercise (HIIE) performed at different times of the day. Based on the morningness–eveningness questionnaire, 12 morning-types (M-types; *N* = 12; age 21 ± 2 years; height 179 ± 5 cm; body mass 74 ± 12 kg) and 11 evening-types (E-types; *N* = 11; age 21 ± 2 years; height 181 ± 11 cm; body mass 76 ± 11 kg) were enrolled in a randomized crossover study. All subjects underwent measurements of Profile of Mood States (POMS), before (PRE), after 12 (POST12) and 24 h (POST24) the completion of both morning (08.00 am) and evening (08.00 p.m.) training. Additionally, Global Mood Disturbance and Energy Index (EI) were calculated. RPE was obtained PRE and 30 min POST HIIE. Two-way ANOVA with Tukey’s multiple comparisons test of POMS parameters during morning training showed significant differences in fatigue, vigor and EI at PRE and POST24 between M-types and E-types. In addition, significant chronotype differences were found only in POST12 after the evening HIIE for fatigue, vigor and EI. For what concerns Borg perceived exertion, comparing morning versus evening values in PRE condition, a higher RPE was observed in relation to evening training for M-types (*P* = 0.0107) while E-types showed higher RPE values in the morning (*P* = 0.008). Finally, intragroup differences showed that E-types had a higher RPE respect to M-types before (*P* = 0.002) and after 30 min (*P* = 0.042) the morning session of HIIE. No significant changes during the evening training session were found. In conclusion, chronotype seems to significantly influence fatigue values, perceived exertions and vigor in relation to HIIE performed at different times of the day. Specifically, E-types will meet more of a burden when undertaking a physical task early in the day. Practical results suggest that performing a HIIE at those times of day that do not correspond to subjects’ circadian preference can lead to increased mood disturbances and perceived exertion. Therefore, an athlete’s chronotype should be taken into account when scheduling HIIE.

**Trial registration**:

ACTRN12617000432314, registered 24 March 2017, “retrospectively registered”.

**Web address of trial**:

https://www.anzctr.org.au/Trial/Registration/TrialReview.aspx?id=371862&showOriginal=true&isReview=true

## Introduction

Chronotype, also defined circadian typology, represents the expression of an individual’s circadian rhythmicity. Three different categories of chronotype can be defined: evening-types (E-types), morning-types (M-types), and neither-types (N-types). Typically, the chronotype is determined with the use of self-assessment questionnaire and the most used is the Morningness–Eveningness Questionnaire (MEQ) by Horne and Ostberg (1976). The existing evidence suggests that chronotype widely affects our biological, behavioral and psychological functions (Adan et al., 2012; Vitale et al., 2017a). M-types, for instance, show an early peak along the day of body temperature (Baehr et al., 2000), serum cortisol (Bailey and Heitkemper, 2001) or blood melatonin circadian rhythm (Mongrain et al., 2004), and they usually perform best in the morning (Rossi et al., 2015). On the contrary, E-types show delayed circadian acrophases compared to M-types in a range of 1–3 h and they have better performances in the evening (Montaruli et al., 2017; Roveda et al., 2017).

The chronotype also influences the individual’s behavioral circadian parameters. A strong association between circadian typology and sleep–wake behavior has been observed (Vitale et al., 2017b): E-types show difficulty in initiating sleep and they usually wake up and go to bed later (Taillard et al., 2004), whereas M-types have early bedtimes, wake up times and show higher objective and subjective sleep quality (Vitale et al., 2015). Furthermore, it is crucial to emphasize that individual differences, meant as the predisposition toward morningness or eveningness, also affect the psychological functioning and the personality (Cavallera and Giudici, 2008). Moods range on a continuum from pleasurable to unpleasant feeling states along the day and it has been demonstrated that M-type women have lower levels of anxiety than E-types and N-types (Muro et al., 2009) whereas evening-oriented people presented higher values of work-related fatigue (Martin et al., 2012). In addition, E-types generally have higher scores in extraversion than M-types (Langford and Glendon, 2002) and a positive relationship between agreeableness/conscientiousness and morningness was observed (Randler, 2008).

Studies on circadian rhythms of perceived exertion, anxiety and mood states in response to physical activity are extremely limited and unclear and it is commonly claimed that mood changes in response to exercise are not influenced by time of day (Trine and Morgan, 1995). It was observed, in male adults, that the state of anxiety, vigor and anger seem to be reduced post-exercise when compared with pre-exercise levels, regardless of the time of day (O’Connor and Davis, 1992; Koltyn et al., 1998), and this result suggests that physical activity can have large effects on mood (Lattari et al., 2014). Noteworthy, it should be stressed that no previous study took into account the subjects’ circadian typology in the study of the psychological responses to exercise at different times of day.

Recently, an association between chronotype, ratings of perceived exertion (RPE), fatigue scores and mood states has been observed (Vitale et al., 2013; Kunorozva et al., 2014). It seems that M-types have more of an advantage in the morning because they are less fatigued in the first hours of the day compared with N- and E-types (Rossi et al., 2015). Rae et al. (2015) reported a significant influence of chronotype on both fatigue and vigor in relation to a maximum-intensity physical task. The authors compared 200-m time-trial swimming performance, RPE and mood state at 06:30 a.m. and 6:30 p.m. in 26 swimmers, classified as 15 M-types and 11 N-types. The Profile of Mood States (POMS) questionnaire, which is a reliable and valid measure of mood in sport settings too (Terry and Lane, 2000), was used to assess the subjects’ affective and mental state (McNair et al., 1971). M-type swimmers reported lower fatigue and higher vigor scores before the 06:30 time trial compared with the 6:30 p.m. and, in addition, they showed lower global mood disturbance (GMD) compared with N-types, irrespective of the time of day.

A recent systematic review deeply examined the effect of chronotype on both the results of, and the psychophysiological responses to, physical activity. The authors concluded that M-types have, in general, both better athletic performances and lower fatigue scores in the morning than N-types and E-types, especially during submaximal and self-paced physical tasks (Vitale and Weydahl, 2017). Nonetheless, few data are available about the chronotype effect on high intensity interval exercises (HIIE). Vitale et al. (2017b) examined, for the first time, actigraphy-based sleep parameters in different chronotypes in relation to two acute sessions of HIIE performed at different times of the day. It was observed that sleep quality was poorer for M-types than E-type soccer players only after the evening training session. In addition, Bonato et al. (2017a,b) highlighted that E-types had a higher peak of salivary cortisol, higher heart rate and higher vagal indices with a significant lower parasympathetic tone respect to M-types when performing a HIIE early in the morning.

It is extremely important to understand the relationship between mood states and physical performance since one variable can influence the other and vice versa. To the best of our knowledge, no previous study examined the chronotype effect on mood in response to HIIE.

Therefore, in the present work, we aimed to study the influence of chronotype on mood state and RPE both before and in response to acute HIIE performed at 08:00 in the morning and at 08:00 in the evening. We hypothesize that M-types have higher vigor scores and lower values of fatigue, depression, anger, tension, mood disturbance and RPE before and after morning exercise than E-types and, on the contrary, that E-types have better psycho-biological responses to HIIE after the evening session.

## Materials and Methods

### Subjects

Sport science student of the School of Sport Science of the Università degli Studi di Milano, Milan, Italy were recruited for the present study during the academic year 2015–2016 (*N* = 547; 389 males and 158 females). Inclusion criteria for subject’s participation to the study were: age ≥18 years; male; being physically active; at least 6 h of training a week and with a morning or evening chronotype scores (see “assessment of circadian typology”). Exclusion criteria were smoking, use of medications and any other medical condition contraindicating physical exercise. Thirty-seven healthy collegiate male students were therefore deemed eligible. Nevertheless, only 24 subjects (12 M-Types and 12 E-Types) agreed to voluntarily participate in the study. Before entering the study, the participants were fully informed about the study aims and procedures, and written informed consent was obtained before testing. The study protocol was approved by the Institutional Ethics Review Committee (approved on 12/10/15, prot. N. 52/15) in accordance with current national and international laws and regulations governing the use of human subjects (Declaration of Helsinki II). This trial was registered at Australian New Zealand Clinical Trials Registry (ACTRN12617000432314). After a baseline anthropometric evaluation, subjects underwent a yo-yo intermittent recovery test level 1 (Bangsbo, 1994) and then they were randomly assigned in a 1:1 ratio according to their chronotype to either morning training (Group A: *N* = 12; age 23 ± 3 years; height 175 ± 7 cm; body mass 73 ± 10 kg, weekly training volume 8 ± 2 h) that started performing the HIIT protocol at 08.00 a.m. or evening training (Group B: *N* = 12; age 21 ± 3 years; height 176 ± 5 cm; body mass 75 ± 11 kg, weekly training volume 8 ± 3 h) that started performing the HIIT protocol at 08.00 p.m. Both groups were blinded about the aim of the study.

### Study Design

**FIGURE 1:**
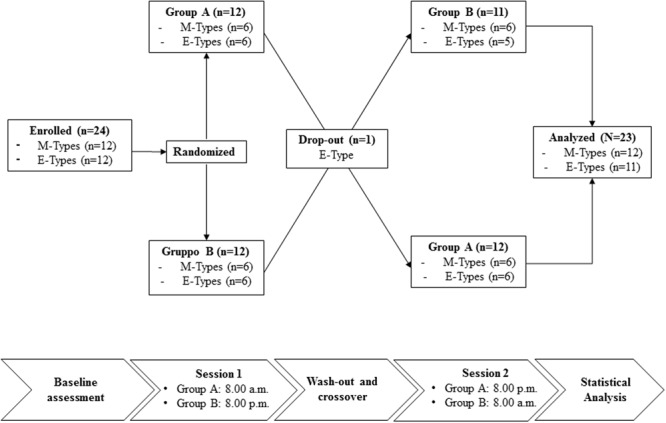
Flowchart for the study design.

This was a randomized crossover study which was carried out in spring, between March and April 2016, over a period of 4 weeks. The experimental design consisted of the following: Group A performed the morning training session at 08:00 a.m. while Group B performed the evening training session at 08:00 p.m.; after a recovery period of 7 days during which subjects maintained their habitual lifestyle without performing physical training, Group A trained in the evening while Group B trained in the morning. The study flowchart is illustrated in **Figure 1**.

One participant in Group B was excluded from analysis because he did not perform the second training session. In each test session measurement of RPE, psychophysiological recovery, and POMS were performed. RPE was performed before and after 30 min HIIT. Measurements regarding psychophysiological recovery were performed after 12 and 24 h after HIIT. All subjects were previously familiarized with all testing procedures.

### Procedures

#### Assessment of Subject’s Circadian Typology

Participants’ circadian typology was assessed by the Horne-Ostberg MEQ (Horne and Ostberg, 1976). According to the MEQ-score, participants were categorized as Morning-type (scoring ≥ 59); Evening-type (scoring ≤ 41) and Neither-type, scoring (42–58). Individual chronotype scores and categories were communicated to the participants only after the completion of the experimentation.

#### Anthropometric Assessment

Anthropometric variables included body mass and stature. Stature was measured with a stadiometer and body mass with a portable scale to the nearest 0.5 cm and 0.1 kg, respectively (Seca 217, Vogel & Halke, Hamburg Germany). Body mass index (BMI) was calculated using the standard formula.

#### Yo-Yo Intermittent Recovery Test Level 1

The Yo-Yo intermittent recovery test Level 1 (Yo-Yo IR1) consisted of repeated 2 × 20-m runs back and forth between the starting, turning, and finishing line at a progressively increasing speed controlled by audio bleeps from a tape recorder (Bangsbo, 1994). The test protocol started with 4 running bouts at 10–13 km . h^-1^ (0–160 m) and another 7 runs at 13.5–14.0 km . h^-1^, and thereafter speed was increased with a stepwise 0.5 km . h^-1^ speed increment every 8 running bouts (i.e., after 760, 1080, 1400, 1720-m etc.) until exhaustion. Between each running bout, the subjects had a 10 s active rest period, consisting of 2 × 5-m of jogging. When the subjects failed twice to reach the finishing line in time, the distance covered was recorded and represented the test result. Tests were performed on the field of an outdoor 400-m track, marked by cones, (1.22 m width and a 20-m length). Another cone placed 5 m behind the finished line marked the running distance during the active recovery period. All tests were conducted from 11:00 a.m. to 02:00 p.m. which is considered an intermediate time of day, and in dry, windless weather conditions with a temperature of about 15–20°C. Before the test, all subjects performed a standardized warm-up at the speed of the first four running bouts of the test. The total duration of the test was 6–20 min. All subjects were previously familiarized with the test, by at least one pre-test. Heart rate was recorded beat-to-beat using a Polar RS800 heart rate monitor (Polar, Kempele, Finland) in order to measure directly the HR_peak_ reached during the test.

#### High-Intensity Interval Exercise Protocol

The HIIE protocol consisted of 4 bouts of 4 min at 90–95% HR_peak_ with 3 min of active recovery at 50–60% HR_peak_ (Helgerud et al., 2001). The calculation of the training percentages was carried out using the HR_peak_ achieved during the Yo-Yo IR1. The training intervention started with a standardized 10-min warm-up and ended with a 3-min cool-down period at a self-selected intensity. Before, during, and after the test HR was recorded beat-to-beat using a Polar RS800 heart rate monitor (Polar, Kempele, Finland). All training sessions were conducted on an outdoor 400 m track in dry, windless weather conditions with a temperature of about 15–20°C. All subjects completed training sessions without complications. The high-intensity endurance interval training protocol was generally well tolerated and subjects did not report dizziness, light-headiness or nausea, symptoms that occasionally occur during this type of training.

#### Rating of Perceived Exertion

The Borg CR-10 category-ratio scale was selected to rate the perceived intensity of exertion (Borg, 1998). A verbal-anchored scale was shown to the subjects before (PRE), and after 30-min (POST) completing HIIE. Each subject was familiarized with the Borg CR-10 scale, including anchoring procedures.

#### Psychological Profile Monitoring

To evaluate the POMS a validated 32-item Italian version (Piacentini et al., 2009) questionnaire reflecting the individuals, mood on five primary dimensions (i.e., depression, fatigue, vigor, tension, and anger) was administered. Athletes were required to describe their mood (depression, fatigue, vigor, tension, and anger) using a 5-point scale (i.e., not at all = 0; somewhat = 1; moderately so = 2; very much so = 3; very very much so = 4). The questionnaires were completed individually, PRE, POST 12 and POST 24 h HIIE. An investigator was present to provide assistance if required. The POMS yields measures of depression, fatigue, vigor, anger and tension. Data were analyzed separately for each specific dimension. Additionally, GMD was calculated by subtracting the vigor score from the sum of the scores of the four remaining subscales. To prevent a negative score, a constant of 100 was added to the global score, in accordance with Morgan et al. (1987). Given that vigor and fatigue are scores that show the greatest changes in response to training (Meeusen et al., 2013), the “energy index” (vigor-fatigue) was used to monitor these changes (Raglin et al., 1991).

### Statistical Analysis

Descriptive statistics (mean ± SD) for the outcome measures were calculated. The normality of the distribution of the anthropometric (weight, height, and BMI), background (age, training hours per week, and years of practice), and Yo-Yo IR1 (total distance and HR_peak_) variables were checked using graphical methods and the D’Agostino Pearson test. Since all variables were normally distributed, differences between Group A and Group B were checked using an unpaired Student’s *t*-test. Parametric statistical tests were also applied to compare the POMS parameters and Borg perceived exertions, when the hypothesis of Gaussian distribution could be assumed. Specifically, intra- and inter-group differences between M-types and E-types were checked using 2-way analysis of variance with Tukey’s multiple comparisons test. A paired *t*-test was used to compare Borg perceived exertions between morning and evening training in both PRE and POST conditions for M-types and E-types. The level of statistical significance was set at *P* < 0.05. Statistical analysis was performed using GraphPad Prism version 6.00 for Mac OSX (GraphPad Software, San Diego, CA, United States). Standardized changes in the mean values were used to assess magnitude of effects (Effect Size, ES). Values < 0.2, <0.6, <1.2 and >2.0 were interpreted as trivial, small, moderate, large and very large, respectively (Batterham and Hopkins, 2006).

## Results

Of the total of 547 subjects (71.1% males and 28.9% females), 345 were N-types (63.1%, 250 males and 95 females), 157 E-types (28.7%, 117 males and 40 females), and 45 M-types (8.2%, 22 males and 23 females). The mean MEQ score, for the whole group, was 47.2 ± 11.5 with a median of 47, 1st quartile of 38.75 and 3rd quartile of 56. The subgroup of 24 subjects was composed by 12 M-types (all moderate M-types) and 12 E-types (3 extreme E-types and 9 moderate E-types). The mean MEQ scores for the subsamples of M-types and E-types were, respectively, 31 ± 3 and 63 ± 3.

**Table 1** reports the pre-HIIE parameters of the 23 subjects divided in Group A (*N* = 12) and Group B (*N* = 11), respectively. Un-paired *t*-test showed that groups were equally matched, showing no significant differences in age, height, body mass, BMI, MEQ-Score and weekly training volume.

**Table 1:** Subjects’ characteristics at baseline.

Parameter	Group A	Group B	*p*	ES
Age (years)	23 ± 3	21 ± 3	n.s.	0.6
Height (cm)	175 ± 7	176 ± 5	n.s.	<0.2
Body Mass (kg)	73 ± 10	75 ± 11	n.s.	0.2
BMI (kg . m^-2^)	23 ± 2	23 ± 3	n.s.	<0.2
MEQ-score (points)	45 ± 16	43 ± 16	n.s.	0.2
Weekly training volume (hours . week^-1^)	8 ± 2	8 ± 3	n.s.	<0.2

*Values are expressed as mean ± SD. MEQ, morningness eveningness questionnaire; ES: Effect Size.*

### Chronotype Effect: M-Types vs. E-Types

#### Morning HIIE

**Table 2** shows the two-way ANOVA with Tukey’s multiple comparisons test with associated *P*-values of POMS parameters during morning HIIE. A significant interaction at PRE and POST24 for fatigue, vigor and EI, with differences between M-types and E-types was found. No significant interactions were found for depression, tension, anger and GMD.

**Table 2:** Results of the 2-way analysis of variance with Tukey’s multiple comparisons test of the seven POMS parameters during morning HIIE for M-types and E-types at PRE, POST12 and POST24.

POMS parameters	PRE	POST 12	POST 24	Interaction	Effect of time	Chronotype effect	Intergroup differences
Depression	*M:* 9.1 ± 1.4 *E:* 10.1 ± 2.2	*M:* 9.4 ± 2.6 *E:* 8.2 ± 0.4	*M:* 9.0 ± 2.7 *E:* 8.4 ± 0.9	n.s.	n.s.	n.s.	PRE: *P* = n.s., ES = 0.7 POST 12: *P* = n.s., 0.5 POST 24: *P* = n.s., 0.2
Fatigue	*M:* 7.6 ± 2.6 *E:* 13.6 ± 5.6	*M:* 10.1 ± 3.4 *E:* 10.2 ± 5.6	*M:* 8.1 ± 2.8 *E:* 13.4 ± 4.8	0.028	n.s.	0.002	PRE: *P* = 0.024, ES > 2.0 POST 12: *P* = n.s., ES < 0.2 POST 24: *P* = 0.045, ES = 1.9
Vigor	*M:* 17.4 ± 4.9 *E:* 12.0 ± 2.1	*M:* 13.8 ± 4.6 *E:* 13.4 ± 2.5	*M:* 15.7 ± 3.8 *E:* 10.2 ± 3.5	0.048	n.s.	0.0002	PRE: *P* = 0.023, ES = 1.1 POST 12: *P* = n.s., ES < 0.2 POST 24: *P* = 0.011, ES = 1.4
Tension	*M:* 8.5 ± 2.7 *E:* 8.2 ± 2.8	*M:* 7.6 ± 3.9 *E:* 6.3 ± 0.7	*M:* 7.5 ± 4.2 *E:* 6.8 ± 1.6	n.s.	n.s.	n.s.	PRE: *P* = n.s., ES < 0.2 POST 12: *P* = n.s., ES = 0.4 POST 24: *P* = n.s., ES = 0.6
Anger	*M:* 8.8 ± 2.9 *E:* 9.4 ± 2.7	*M:* 9.8 ± 2.9 *E:* 7.3 ± 0.7	*M:* 9.5 ± 5.1 *E:* 9.0 ± 1.9	n.s.	n.s.	n.s.	PRE: *P* = n.s., ES = 0.2 POST 12: *P* = n.s., ES = 0.8 POST 24: *P* = n.s., ES < 0.2
Global mood disturbance	*M:* 33.1 ± 4.7 *E:* 32.7 ± 5.7	*M:* 32.9 ± 4.2 *E:* 29.1 ± 6.1	*M:* 32.4 ± 7.9 *E:* 30.2 ± 5.1	n.s.	n.s.	n.s.	PRE: *P* = n.s., ES < 0.2 POST 12: *P* = n.s. ES = 0.9 POST 24: *P* = n.s., ES = 0.3
Energy Index	*M:* 9.7 ± 6.5 *E:* -1.2 ± 7.7	*M:* 2.7 ± 6.8 *E:* 3.2 ± 6.4	*M:* 8.1 ± 5.1 *E:* -2.1 ± 8.7	0.015	n.s.	0.0002	PRE: *P* = 0.006, ES = 1.7 POST 12: *P* = n.s., ES < 0.2 POST 24: *P* = 0.012, ES = 2.0

*M, M-types; E, E-types; n.s., not significant; ES, Effect Size.*

#### Evening HIIE

**Table 3** shows the two-way ANOVA with Tukey’s multiple comparisons test with associated *P*-values of POMS parameters during evening HIIE. A significant interaction at POST12 for fatigue, vigor and EI, with differences between M-types and E-types was found. No significant interactions were found for depression, tension, anger and GMD.

**Table 3:** Results of the 2-way analysis of variance with Tukey’s multiple comparisons test of the seven POMS parameters during evening HIIE for M-types and E-types at PRE, POST12 and POST24.

POMS parameters	PRE	POST 12	POST 24	Interaction	Effect of time	Chronotype effect	Intergroup differences
Depression	M: 11.0 ± 3.7 E: 8.8 ± 2.4	M: 9.4 ± 2.9 E: 8.9 ± 1.9	M: 9.3 ± 2.4 E: 8.1 ± 0.3	n.s.	n.s.	n.s.	PRE: *P* = n.s., ES = 0.5 POST 12: *P* = n.s., ES = 0.2 POST 24: *P* = n.s., ES = 0.5
Fatigue	M: 8.2 ± 1.6 E: 9.7 ± 4.4	M: 8.4 ± 1.8 E: 12.9 ± 4.8	M: 10.1 ± 3.0 E: 8.1 ± 2.1	0.005	n.s.	n.s.	PRE: *P* = n.s., ES = 0.9 POST 12: *P* = 0.019, ES > 2.0 POST 24: *P* = n.s., ES = 0.6
Vigor	M:15.8 ± 3.8 E: 15.0 ± 2.8	M: 16.1 ± 3.4 E: 10.8 ± 4.4	M: 12.9 ± 4.4 E: 14.6 ± 2.9	0.009	n.s.	n.s.	PRE: *P* = n.s., ES = 0.2 POST 12: *P* = 0.019, ES = 1.5 POST 24: *P* = n.s., ES = 0.4
Tension	M: 8.5 ± 3.9 E: 7.4 ± 2.5	M: 7.2 ± 1.7 E: 7.1 ± 2.2	M: 8.3 ± 3.4 E: 6.2 ± 0.4	n.s.	n.s.	n.s.	PRE: *P* = n.s., ES = 0.3 POST 12: *P* = n.s., ES < 0.2 POST 24: *P* = n.s., ES = 0.6
Anger	M: 8.3 ± 1.4 E: 8.2 ± 2.9	M: 8.0 ± 1.3 E: 10.0 ± 5.3	M: 9.0 ± 2.1 E: 7.5 ± 1.0	n.s.	n.s.	n.s.	PRE: *P* = n.s., ES < 0.2 POST 12: *P* = n.s., ES = 1.5 POST 24: *P* = n.s., ES = 0.7
Global mood disturbance	M: 29.8 ± 4.9 E: 31.5 ± 5.0	M: 30.3 ± 4.1 E: 31.9 ± 6.6	M: 31.0 ± 6.3 E: 28.4 ± 3.0	n.s.	n.s.	n.s.	PRE: *P* = n.s., ES = 0.3 POST 12: *P* = n.s., ES = 0.4 POST 24: *P* = n.s., ES = 0.4
Energy Index	M: 7.6 ± 4.7 E: 5.2 ± 6.2	M: 7.7 ± 4.2 E: -2.1 ± 8.8	M: 2.8 ± 5.9 E: 6.5 ± 4.1	0.0014	n.s.	n.s.	PRE: *P* = n.s., ES = 0.5 POST 12: *P* = 0.003, ES > 2.0 POST 24: *P* = n.s., ES = 0.6

*M: M-types; E: E-types; n.s.: not significant; ES: Effect Size.*

**FIGURE 2:**
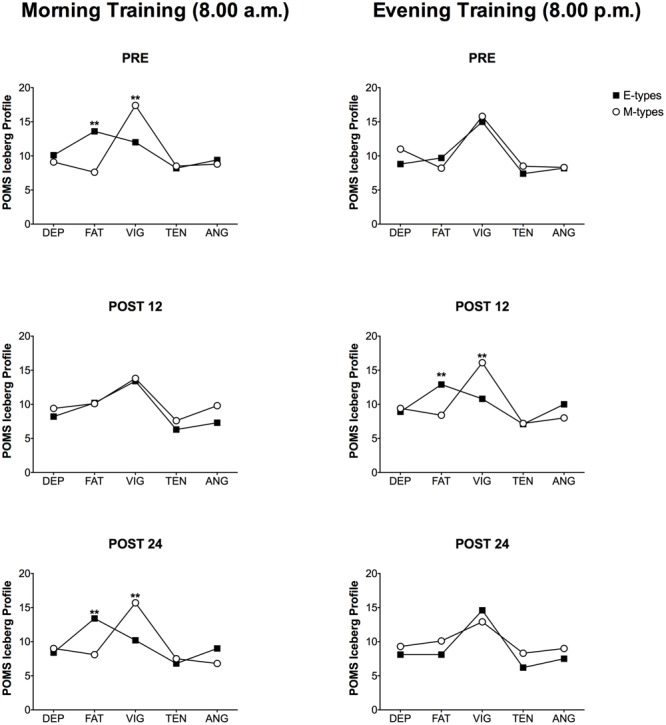
Profile of Mood States (POMS) Iceberg Profiles of the five POMS parameters during morning and evening HIIE for M-types and E-types at PRE, POST12 and POST24. DEP, depression; FAT, fatigue; VIG, vigor; TEN, tension; ANG, anger; ^∗∗^*P* < 0.01. Please note that the comparison between M-types and E-types refers to the statistical analysis reported in **Tables 2, 3**.

**FIGURE 3:**
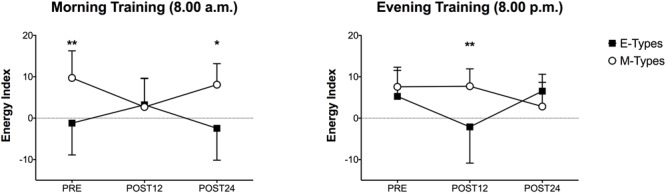
Energy Index (EI) values during morning and evening HIIE for M-types and E-types at PRE, POST12 and POST24. ^∗^*P* < 0.05; ^∗∗^*P* < 0.01. Please note that the comparison between M-types and E-types refers to the statistical analysis reported in **Tables 2, 3**.

**Figure 2** shows the POMS Iceberg Profile in relation to morning and evening training, whereas **Figure 3** refers to the EI values of both training sessions. The significant differences reported in **Figures 2, 3** are based on 2-way analysis of variances, showed in **Tables 2, 3**.

#### Rating of Perceived Exertion

**FIGURE 4:**
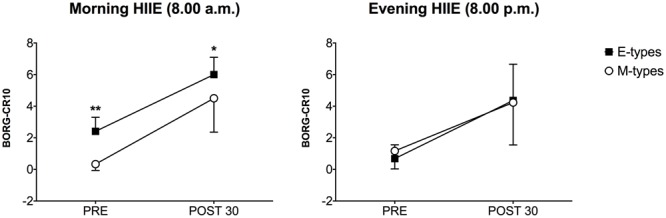
Ratings of perceived exertion (RPE) during morning and evening HIIE for M-types and E-types at PRE and POST30. ^∗^*P* < 0.05; ^∗∗^*P* < 0.01.

Comparing morning versus evening PRE HIIE values, a higher RPE was observed in the evening for M-types (0.3 ± 0.3 vs. 1.7 ± 1.1, *P* = 0.0107, ES = 1.2) while, conversely, E-types reported higher RPE values before the start of the morning training session (2.4 ± 0.9 vs. 0.7 ± 0.9, *P* = 0.008, ES = 1.8) (**Figure 4**). No significant differences in POST condition were detected. Furthermore, as expected, RPE increased significantly 30 min post HIIE for both morning (M-types: 0.3 ± 0.4 vs. 4.5 ± 2.1, *P* < 0.0001, ES > 2.0; E-Types: 2.4 ± 0.8 vs. 6.0 ± 1.1, *P* < 0.0001, ES > 2.0) and evening (M-types: 1.2 ± 1.1 vs. 4.2 ± 2.7, *P* = 0.001, ES > 2.0; E-Types: 0.7 ± 0.9 vs. 4.4 ± 2.3, *P* = 0.001; ES > 2.0) training bouts. In conclusion, intragroup differences showed that during morning HIIE E-types had a higher RPE respect to M-types before (2.4 ± 0.9 vs. 0.3 ± 0.3, *P* = 0.002, ES > 2.0) and after 30 min (6.0 ± 1.1 vs. 4.5 ± 2.1, *P* = 0.042, ES = 1.6) HIIE. No significant changes during evening HIIE were found.

## Discussion

The main finding of the present study is that both RPE and mood states responses to an acute session of HIIE performed at different times of the day, are influenced by the subject’s chronotype. Specifically, E-types are more fatigued, show less vigor and perceive more exertion in relation to a morning session of HIIE compared both to the evening training session and with M-types. On the contrary, mood state and scores of perceived exertions did not vary in relation to the evening session of HIIE, only M-types reported higher RPE at 08:00 p.m. compared to their morning values. It seems that performing a HIIE in the first hours of the day could generate mood disturbances and negatively influence the psychophysiological responses to physical activity for E-types but not for M-types.

It is known that morningness scores tend to increase with age (Merikanto et al., 2012) and that males are significantly more evening-oriented than females (Adan et al., 2012). Since we recruited young college students with a large predominance of males (71.1%), we observed, as expected, a larger number of E-types (28.7%) than M-types (8.2%). These results are totally in line with previous studies that investigated the chronotype distribution among young students (Adan et al., 2012; Vitale et al., 2015). One of the strengths of this work is the clear homogeneity of the sample. The participants recruited were 23 healthy and physically active male college students, categorized in 12 M-types and 11 E-types, and they were totally comparable for age, height, weight, BMI and weekly training volume, both when randomly grouped in group A and group B (**Table 1**) and also when divided for chronotype category.

Despite the literature concerning the chronotype effect on athletic performance and the psychophysiological responses to physical activity has increased over the last years, there are still few and conflicting results. A recent systematic review highlighted that M-types have better athletic performances in the morning compared to other chronotypes (Vitale and Weydahl, 2017) but, most of all, the more evident results can be observed for RPE and fatigue scores in relation to physical activity. (Brown et al., 2008; Henst et al., 2015; Rae et al., 2015) Previous studies showed that M-types perceived less exertion when performing a moderate-intensity physical task in the morning, while E-types showed higher fatigue values in the first part of the day (Vitale et al., 2013; Kunorozva et al., 2014; Rae et al., 2015; Rossi et al., 2015).

In particular, before the start of the morning HIIE session, we observed that E-types had significantly higher RPE (2.4 ± 0.8) both than M-types (0.3 ± 0.4) and their evening values (0.7 ± 0.9). Moreover, also RPE values post HIIE performed at 08:00 remained markedly higher for E-types (6.0 ± 1.1) compared with morning subjects’ scores (4.5 ± 2.1). The only difference observed in relation to the evening HIIE session is that M-types reported higher RPE values in the PRE condition, (1.2 ± 1.1) compared to their morning values (0.3 ± 0.4). The same trend was reported by Kunorozva et al. (2014): they noted that M-type cyclists had higher RPE when cycling at 18:00 and 22:00 compared to the morning sessions. Furthermore, Rossi et al. (2015) highlighted that E-type college students had higher RPE at 08:30 in response to a self-paced walking task compared with M-types.

To confirm this, the results of POMS questionnaire are on the same line. We observed a significant chronotype effect on vigor, fatigue and Energy Index (EI) scores: M-types had higher vigor (17.4 ± 4.9) and energy (9.7 ± 6.5) and lower fatigue (7.6 ± 2.6) than E-types (vigor: 12.0 ± 2.1; EI: -1.2 ± 7.7; fatigue: 13.6 ± 5.6) before performing the morning physical task. Nonetheless, anger, depression, tension and GMD scores did not vary according to the subjects’ circadian typology. An interesting result is that the same differences were observed the morning after (POST 24), but not in the evening of the same day (POST 12) (**Figure 2** and **Table 2**). To avoid any factor that could confound the mood states the subjects were asked to lead their following day, meant as the 24 h post exercise, with their normal habits and without any kind of physical activity to not influence the POMS results. No significant chronotype effect on the POMS items was observed in PRE and POST 24 HIIE evening session (both time periods refer to evening hours). However, curiously, the morning after (POST 12), E-types reported lower vigor and EI scores and they were more fatigued compared to M-types (**Figure 2** and **Table 3**).

To the best of our knowledge, Rae et al. (2015) conducted the first and only study that evaluated the effect of chronotype on POMS items in relation with a physical task performed at different times of the day. Their results are in line with the present study: no differences were observed for GMD in accordance with the chronotype group but a significant interaction time-by-chronotype was detected for the sub-items: M-types had lower fatigue and higher vigor scores prior to the morning physical test compared to the evening session. Therefore, the lower perception of effort and greater vigor in the morning for M-types may lead them to reach better performances in the first part of the day.

All these results highlight the fact that, in general, the early hours of the day seem to represent a time that could create more disadvantage in the psychophysiological responses to HIIE, especially for E-types. Previous studies remarked this concept and reported a chronotype effect on HIIE too. Bonato et al. (2017a,b) showed that E-types had higher morning levels of salivary cortisol, heart rate and presented a significant parasympathetic withdrawal with a sympathetic predominance respect to M-types when performing a HIIE at 08:00 a.m. whereas the same differences were not observed in the evening. Furthermore, on the contrary, Vitale et al. (2017b) reported that an evening session of high intensity interval training is more suitable for E-type collegiate soccer players: sleep quality, evaluated through actigraphy, was poorer in M-types than in evening-oriented subjects in response to the evening HIIE session.

This investigation has a number of limitations that should be discussed. First, the study population was composed by a relatively small sample and no power calculations were performed. The participants are representative only of male athletes practicing soccer while female athletes and other sports disciplines were not considered. Second, it is essential, in future studies, to control of potential confounders: it will be necessary to make appropriate decisions when selecting between field-based or laboratory-based performance tests and the differences between training sessions and official competitions should be considered.

## Conclusion

Chronotype seems to significantly influence the psychophysiological responses to physical activity. Fatigue, perceived exertion and vigor in relation to HIIE performed at different times of the day are affected by the subjects’ circadian typology. Specifically, E-types will meet more of a burden when undertaking a physical task early in the day. Practical results suggest that performing an HIIE at those times of day that do not correspond to subjects’ circadian preference can lead to increased mood disturbances and perceived exertion. Therefore, an individual’s chronotype should be taken into account by conditioning coaches when scheduling HIIE.

## Author Contributions

JV, ALT, RB, MFP, and MB, substantially contribute to the conception and in the design of the work, made the acquisition, analysis, and interpretation of data for the work. In addition, they drafted the work and revised it critically for important intellectual content. Moreover, they made the final approval of the version to be published and made the agreement for all aspects of the work in ensuring that questions related to the accuracy or integrity of any part of the work are appropriately investigated and resolved.

## Conflict of Interest Statement

The authors declare that the research was conducted in the absence of any commercial or financial relationships that could be construed as a potential conflict of interest.
